# Erythrophlæine as a Local Anæsthetic

**Published:** 1888-04

**Authors:** Carl Koller

**Affiliations:** Vienna


// The Medical Journal and Examiner.

VOLUME LVI. APRIL, 1888. NUMBER 4.

### ERYTHROPHLÆINE AS A LOCAL ANÆSTHETIC.

BY CARL KOLLER, M. D.

Lewin, of Berlin,* has lately directed public attention to erythrophlæine, an alkaloid contained in the bark of Erythrophlacum Guinense, which also forms an ingredient of the African "Haya-poison." Apart from the pharmacological and physiological interest which these researches possess, universal consideration is due to Lewin's statement that erythrophlæine possesses in a high degree the quality of producing local anæsthesia of a duration of several hours—even of one to two days.

Since I, about three years ago, introduced cocaine, the first local anæsthetic, into therapeutics, many endeavors have been made to discover among the vegetable bases such as possess the same or still higher anæsthetic properties; indeed, some substances have become known, which may correctly be called local anæsthetics, but whose employment in practice was hindered by some undesirable secondary effects.

I occupied myself in some experiments to try the effect of erythrophlacinum hydrochloricum on the eye, a small quantity of this substance having been placed at my disposal by Mr. E. Merck, of Darmstadt.

* A paper read in the Berliner Medicinische Gesellschaft, January 11, 1888.

The instillation of two drops of freshly prepared 0.25 per cent. solution in the eye of a dog produces the following appearances: About one minute after the application a frequent blinking with the eyelids begins, evidently in consequence of pain that still increases, for with its growing uneasiness the animal tries to wipe its eye with its paw or to rub it against some object. The eye remains closed as in a spasm; the conjunctiva is very red; injection of ciliary vessels is present. After about 20 minutes the irritation seems to reach its climax; at this time both eyes are kept closed. After another five minutes the eye is opened for a while, then for a longer time, until finally about half an hour after the beginning of the experiment it remains open. The period of irritation is passed; the conjunctiva is only slightly injected. The cornea is proven to be now quite insensible to touch and pin-pricks, which insensibility lasts at least some hours. No influence upon the pupil was observed. On the next day the eye is convulsively closed, the conjunctiva much swollen and reddened, the limbus cornea raised; the surface of the cornea is clouded, so that the pupil is hardly visible; 48 hours after the beginning of the experiment the cloudiness of the cornea was unchanged; after 72 hours the cloudiness was clearing, but had not entirely disappeared. I convinced myself by repeated experiments that the corneal cloudiness takes place, even if the trial of sensibility is avoided, which is always an ill treatment of the cornea.

Upon myself I experimented only once, and that with a 0.125 per cent. solution. In from one to two minutes after I had instilled two drops into my eye I felt a violent burning; at the same time the conjunctiva became injected and the eye began to water; the burning increased, and with simultaneous reddening of the skin the pain radiated into the whole corresponding half of the face, also into the ear, but especially into the nose. The pain and other symptoms of irritation reached their climax about 20 minutes after the beginning of the experiment; then diminished slowly, and only disappeared entirely after 35 to 40 minutes, calculated from the beginning.

The cornea was now quite anæsthetic; the sensibility to touch was already diminished in the second half of the irritation period. The anæsthesia lasted several hours in the same degree, and even on the following morning the sensibility to touch was diminished. In the pupil and the accommodation no alteration was to be observed, except, perhaps, a slight contraction of the pupil accompanying the irritation and corresponding to its intensity. About an hour and a half after the beginning of the experiment the sight became troubled; a thin mist lay on the objects, and as cause of that, a cloudiness of the corneal ephithelium was to be observed; the eye was without brightness. The dimness toward the evening of the same day increased so that medium-sized printing could not be read. Around all light flames the phenomenon of a spectral ring appeared, blue inside, red outside, so well known from the symptomatology of glaucoma attacks. On the following morning the cloudiness was a little less, but lasted the whole day and had only disappeared the next morning.

Of the appearances described, two seem to me to be of especial interest—the symptoms of irritation and the clouding of the cornea. All local anæsthetics hitherto known irritate in the beginning of their action, and, indeed, it is to be understood that the process by which finally the peripheral ends of nerves become paralyzed is felt as an irritation. With erythrophlæine the period of irritation is comparatively a long one. While after application of a 2 per cent. or stronger solution of cocaine it lasts about a minute, its duration after application of an $\frac{1}{8}$ per cent. eyrthrophlæine solution is longer than one-half hour. Corresponding to that, also, the influence on the nerves goes much farther, as can be recognized from the long duration of the corneal anæsthesia—many hours as against 10 to 15 minutes with cocaine. It might be supposed that danger could arise from this deep alteration of the nerves.

The clouding of the corneal epithelium is an appearance which occurs also in the use of cocaine, although in a very slight degree, and generally hardly visible, except in the rare cases in which cocaine used for operations was accused of having caused a cloudiness of the cornea of long duration. I myself, in the beginning, considered this appearance as a consequence of evaporation or drying from widely opened eyelids, enlarged eyelid-split, and insufficient winking, and I convinced myself by experiment that evaporation plays a part. But the evaporation can not be the only cause of the clouding, for, also, if it had been quite excluded, one would yet observe a slight epithelium cloudiness and point-like epithelium defects. First, my experiments with erythrophlæine strengthen me in my supposition that the cloudiness appearing with or after the anæsthesia, and increasing afterward, arises directly from the influence of these poisons. The relation existing between the duration of the irritation period, the duration of anæsthesia, and the intensity of the cloudiness clearly points to it. To explain that clouding, one must not think of an action like that of a corrosive, but of a subtler one, by which, in the epithelial cells, either directly or in consequence of paralyzing of the nerves, a trouble of nutrition is caused and leads to the clouding.

To the subcutaneous injection of an erythrophlæine solution succeeds likewise a period of pain of a certain duration, and then apparent local anæsthesia of the skin; but this is difficult to be ascertained with animals, and I abstained from experiments upon persons on account of the intense virulence of the drug. A dog, 6 kilogrammes in weight, into which I had made an injection of 0.002 erythrophlæine, vomited violently.

Whether the local anæsthetizing power of erythrophlæine alone or in suitable combination with cocaine may be advantageously used in therapeutics can only be proved by further experiments.

VIENNA, February, 1888.

